# Angiopoietin-2: A Potential Mediator of the Glycocalyx Injury in Adult Nephrotic Patients

**DOI:** 10.3390/jcm7110401

**Published:** 2018-10-31

**Authors:** Maria Moura Santana Chaves, Matheus de Souza Mendes, Maximilian Pinho Schwermann, Raquel Queiroz, Regina Freitas Coelho, Francisco Thiago Santos Salmito, Gdayllon Cavalcante Meneses, Alice Maria Costa Martins, Ana Cristina de Oliveira Monteiro Moreira, Alexandre Braga Libório

**Affiliations:** 1 Medical Sciences Postgraduate Program, Universidade de Fortaleza–UNIFOR, Fortaleza 60811-905, Ceara, Brazil; santanafrota@gmail.com (M.M.S.C.); acomoreira@unifor.br (A.C.d.O.M.M.); 2 Medical Course, Universidade de Fortaleza—UNIFOR, Fortaleza 60811-905, Ceara, Brazil; mthsdsz@gmail.com (M.d.S.M.); maximilian.pinho@gmail.com (M.P.S.); raquel.queirozsl@hotmail.com (R.Q.); regina_rfc@hotmail.com (R.F.C.); 3 Department of Clinical Medicine, Universidade Christus, Fortaleza 60811-905, Ceara, Brazil; thiagosalmito@yahoo.com.br; 4 Medical Sciences Postgraduate Program, Department of Clinical Medicine, Universidade Federal do Ceará, Fortaleza 60811-905, Ceará, Brazil; gdayllon@yahoo.com.br; 5 Department of Clinical and Toxicological Analysis, Faculty of Pharmacy, Federal University of Ceara, Fortaleza 60811-905, Ceara, Brazil; martinsalice@gmail.com; 6 Experimental Biology Centre (NUBEX), University of Fortaleza (UNIFOR), Fortaleza 60811-905, Ceara, Brazil

**Keywords:** Agiopoietin-2, mediation analysis, nephrotic syndrome

## Abstract

Introduction: Glomerulopathy is a group of diseases that affect mainly young adults between the ages of 20 and 40 years. Recently, it has been demonstrated that syndecan-1, a biomarker of endothelial glycocalyx damage, is increased in nephrotic patients with near-normal renal function and it is important to endothelial dysfunction in these patients. Angiopoietin-2 (AGPT2) is an endothelial growth factor that promotes cell derangement. Here we evaluated AGPT2 levels in patients with nephrotic syndrome, near-normal renal function and the possible interaction of AGPT2 with endothelial glycocalyx derangement. Methods: This was a cross-sectional study performed from January through November 2017. Adult patients (age > 18 years) with nephrotic syndrome and without immunosuppression were included. Blood samples were drawn after a 12 h fast for later measurement of syndecan-1 and AGPT2. Mediation analyses were performed to assess the hypothesized associations of nephrotic syndrome features and AGPT2 with syndecan-1. Results: We included 65 patients, 37 (56.9%) of them female, with primary glomerular disease. Syndecan-1 in nephrotic patients was higher than in control individuals (102.8 ± 36.2 vs. 28.2 ± 9.8 ng/mL, *p* < 0.001). Correlation of syndecan-1 with the main features of nephrotic syndrome after adjustment for age and estmmated glomerular filtration rate (eGFR) demonstrated that syndecan-1 was significantly associated with 24-h urinary protein excretion, total cholesterol, LDL (low density lipoprotein)-cholesterol, HDL (high-density lipoprotein)-cholesterol, and triglycerides. Angiopoietin-2 was independently associated with serum albumin, 24 h urinary protein excretion, total cholesterol, and LDL-cholesterol, in addition to being strongly associated with syndecan-1 (0.461, *p* < 0.001). The results of the mediation analyses showed that the direct association between LDL-cholesterol and syndecan-1 was no longer significant after AGPT-2 was included in the mediation analysis. AGPT2 explained 56% of the total observed association between LDL-cholesterol and syndecan-1. Conclusion: The association between LDL-cholesterol and glycocalyx derangement in nephrotic patients is possibly mediated by AGPT2.

## 1. Introduction

Glomerulopathy is a group of diseases that mainly affect young adults between the ages of 20 and 40 years. Patients with nephrotic syndrome generally present with serious edema, lipid alterations, hypoalbuminemia, and possible loss of renal function [1]. The primary alteration in nephrotic syndrome is a loss of the glomerular filtration barrier, consisting of the glomerular endothelium, glomerular basement membrane, and podocytes. One component of the filtration barrier that sustains major damage in nephrotic syndrome is the podocyte. Podocytes are highly differentiated epithelial cells that form a complex molecular network known as the slit diaphragm, which is pivotal for maintaining the size-selective nature of the glomerular filtration barrier [2,3].

Nephrotic patients have an increased relative risk of cardiovascular events, resulting in an almost 3-fold increase in cardiac mortality [4]. It is a widely held view that impaired endothelial function is the initial step in atherogenesis, which is largely responsible for ischemic heart disease and thrombotic strokes occurring in the later decades of life [5]. Recently, our group demonstrated that syndecan-1, a biomarker of endothelial glycocalyx damage, is increased in nephrotic patients with near-normal renal function and is involved in endothelial dysfunction in these patients [3].

We reported the association of syndecan-1 with several features of nephrotic syndrome, such as the lipid profile and 24 h urinary protein excretion rate. The mechanism of how nephrotic syndrome causes endothelial glycocalyx derangement and resulting endothelial dysfunction remains largely unknown. Angiopoietin-2 (AGPT2) is an endothelial growth factor that promotes polymorphonuclear cell infiltration, induces endothelial cell apoptosis and with angiopoietin-1, modulates endothelial permeability via endothelial cell junctions [6]. Glomerular AGPT2 expression is markedly upregulated in animal models of proteinuric nephropathies [7], leading to apoptosis of glomerular endothelia. In patients with chronic kidney disease, AGPT2 is associated with the level of syndecan-1 and with changes in the endothelial surface layer [8].

In this study, we evaluated AGPT2 levels in patients with nephrotic syndrome, near-normal renal function and the possible interaction of AGPT2 with endothelial glycocalyx derangement. To explore the relationship between nephrotic syndrome features AGPT2 and glycocalyx injury, we used a mediation analysis [9]. Although the cross-sectional design of our study does not give direct evidence of the causal mechanisms that we propose, statistical mediation provided a framework in which we could formally test the observed data for evidence of such mechanisms. A mediation analyses can show whether some or all the significance of the association between an exposure and an outcome can be explained statistically by the effect of the exposure, on the potential mediator [10]. The analysis also estimates the proportion of the observed effect that can be explained by exposure to the mediator, if the hypothesized mechanism exists. Although this evidence is circumstantial, it can still provide important support and motivation for more definitive investigations.

## 2. Methods

### 2.1. Subjects

This was a cross-sectional study performed from January through November 2017. Adult patients (age >18 years) with nephrotic syndrome who had undergone a renal biopsy were included. The diagnosis of nephrotic syndrome was performed when patients had 24 h urinary protein excretion >3.5 g/24 h/1.73 m^2^, edema, hypoalbuminemia, and elevated serum lipids. Patients with a significant reduction in renal function (glomerular filtration rate <60 mL/min) were not included. Glomerular filtration rate (GFR) was estimated using the Chronic Kidney Disease Epidemiology Collaboration (CKD-EPI) equation [11].

Subjects with cardiovascular disease, diabetes, hypothyroidism, liver disease, alcoholism, concurrent diseases, and significant psychiatric disorders were excluded. Patients receiving angiotensin-converting enzyme inhibitors, angiotensin II receptor antagonists, diuretics, or any other antihypertensive drugs were not excluded, as these constitute best clinical practice. 

Because syndecan-1 and AGPT2 do not have an established normal range, a control group was included. The control group comprised subjects with no renal disease or significant comorbidity that were recruited from the community. Subjects were studied while they were not taking lipid-lowering drugs or aspirin. The Institutional Ethical Committee approved this study, and patients signed an informed consent form prior to enrollment.

### 2.2. Laboratory Analysis

Ethylenediaminetetraacetic acid (EDTA) tubes were used to collect blood samples after a 12 h fast. The samples were immediately processed and frozen at −80 °C for later measurement of syndecan-1 and AGPT2. Syndecan-1 was measured as a biomarker of endothelial glycocalyx injury (Human Syndecan 1 ELISA kit cat#ab46506, Abcam, Cambridge, MA, USA). The detection range for syndecan-1 is 8–256 ng/mL and the intra-assay coefficient of variation is 6.2%. Angiopoietin-2 was measured in duplicate using an enzyme-linked immunosorbent assay (Human Angiopoietin 2 ELISA kit DY623; R&D Systems, Minneapolis, MN, USA). The interassay coefficient of variation was 5.3%.

### 2.3. Statistical Analysis

The normality of the data was assessed and data are reported as the mean and standard deviation, or median and interquartile range (IQR; 25th–75th percentiles) as appropriate. Baseline characteristics were compared using a 2-sample *t*-test or Mann-Whitney test for continuous variables, whereas dichotomous variables were assessed with a χ^2^ test or Fisher’s exact test. Simple correlations between continuous variables were analyzed using Spearman’s rank correlation coefficient. Non-normal distributions were natural log-transformed for additional analysis. Multivariable logistic regressions were used to assess the association of syndecan-1 and features of nephrotic syndrome. The model was adjusted for age, gender, glomerular filtration rate, 24 h urinary protein excretion, serum albumin, serum total cholesterol, LDL (low density lipoprotein)-cholesterol, HDL (high-density lipoprotein)-cholesterol, and triglycerides. 

Mediation analyses were performed when appropriate and were based on the logistic regression results to assess the hypothesized associations of nephrotic syndrome features and AGPT2 with syndecan-1. Specifically, mediation analysis was performed when the exposure was significantly correlated to the mediator and outcome (after adjustment for confounders) and when the mediator was significantly correlated to the outcome. Indirect effects and confidence intervals were estimated by bootstrapping with 5000 resamples using the PROCESS Statistical Package for SPSS (PROCESS version 2–note that version 3 does not run dichotomous outcomes and SPSS, version 20.0, 2011; SPSS Inc., Chicago, IL, USA) [12]. Statistically significant mediation was established when the indirect effect was significantly different from zero, with full mediation defined as additional attenuation of the association between independent and dependent variables into non-significance, after inclusion of the mediator variable or variables. All comparisons are two-tailed, with *p* < 0.05 considered significant.

## 3. Results

### 3.1. Patient Characteristics

After all exclusion criteria were applied, 65 patients, 37 (56.9%) female, with primary glomerular disease were included. Glomerular histology, glomerular filtration rate, and other nephrotic syndrome-related variables of the patients are described in Table 1. The mean patient age was 38.1 ± 12.6 years. The mean 24 h urinary protein excretion was in the nephrotic range of 5.9 ± 2.2 g/24 h and serum albumin was decreased to 2.6 ± 0.9 g/dL. The mean glomerular filtration rate was 87.3 ± 19.6 mL/min/1.73 m^2^. Fifty-seven patients were being treated with ACE inhibitors or an angiotensin II receptor antagonist and 36 were taking HMG-CoA reductase inhibitors (statins). No other immunosuppressive therapy was administered, except for prednisone at less than 0.2 mg/kg/day (8 patients). 

### 3.2. Syndecan-1 in Nephrotic Patients 

**Figure 1:**
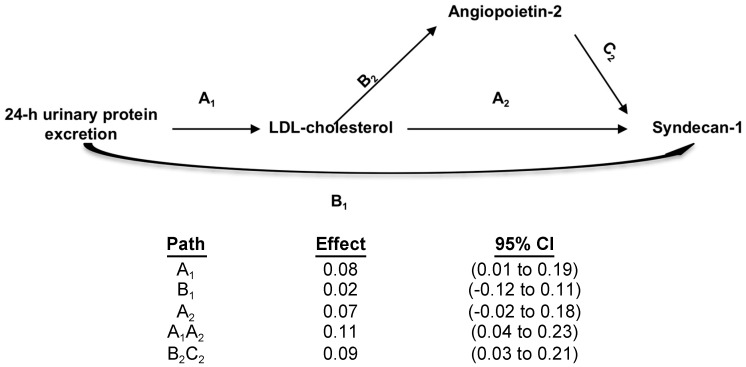
Mediation analyses of the association between LDL-cholesterol and syndecan-1. Path models and mediation analyses describing mediation of the relation between 24 h urinary protein excretion, LDL-cholesterol, syndecan-1, and angiopoietin-2. Path effects are reported as the difference in LDL-cholesterol (mg/dL) observed by per 1-SD difference in 24-h urinary protein excretion and/or in syndecan-1 (ng/mL) observed per 1-SD difference in angipoietin-2, with 95% confidence intervals (95% CIs). Models are adjusted for age, glomerular filtration rate and other nephrotic syndrome features. Residual direct effects are labeled as path A in each model, and indirect effects are labeled as B and C.

The mean value of sndecan-1 in nephrotic patients was 102.8 ± 36.2 ng/mL, which was higher than in control individuals (28.2 ± 9.8 ng/mL, *p* < 0.001). Partial correlation of syndecan-1 with the main features of nephrotic syndrome after adjustments for age and eGFR are presented in Table 2. As demonstrated previously by our group after adjustment for age and glomerular filtration rate, syndecan-1 was significantly associated with 24 h urinary protein excretion, total cholesterol, LDL-cholesterol, HDL-cholesterol, and triglycerides. Interestingly, 24 h urinary protein excretion was not associated with syndecan-1 after adjusting for LDL-cholesterol (*rp* = 0.027, *p* = 0.738), indicating LDL-cholesterol fully mediates 24 h urinary protein excretion effects on syndecan-1 levels (Figure 1). No significant association was observed between serum albumin and syndecan-1. The strongest association of syndecan-1 in these patients was with LDL-cholesterol (*rp* = 0.388, *p* < 0.001). In multivariate analyses, the following parameters were included: Gender, age, glomerular filtration rate, and all nephrotic syndrome features (24 h urinary protein excretion, serum albumin, serum total cholesterol, LDL-cholesterol, HDL-cholesterol, and triglycerides). The only variables independently associated with syndecan-1 were age and LDL-cholesterol (Table 3).

### 3.3. Angiopoietin-2 is Closely Associated with Syndecan-1 and LDL-Cholesterol 

We also investigated the association of AGPT-2 with features of nephrotic syndrome. It was associated with serum albumin, 24 h urinary protein excretion, total cholesterol, and LDL-cholesterol (Table 2). The strongest association was with LDL-cholesterol (*rp* = 0.408, *p* < 0.001).

### 3.4. Mediation Analysis 

To further investigate the role of AGPT-2 in glycocalyx endothelial damage, we evaluated if the association between LDL-cholesterol (the only nephrotic syndrome feature independently associated with syndecan-1) was mediated by AGPT-2. First, we evaluated the association between AGPT2 and syndecan-1 (*r* = 0.461, *p* < 0.001). The results of the mediation analyses are summarized in Figure 1. The direct association between LDL-cholesterol and syndecan-1 was no longer significant after AGPT-2 was included in the mediation analysis. The indirect effect explained 56% of the total observed effect.

## 4. Discussion

To the best of our knowledge, this study reports the first evaluation of the role of AGPT2 in nephrotic patients with a near-normal glomerular filtration rate. We found that nephrotic patients had an increased level of serum AGPT2 in comparison with control subjects. Our data suggest, through mediation analysis, that AGPT2 is involved in the association between nephrotic syndrome features (mainly high LDL-cholesterol) and endothelial glycocalyx derangement. 

AGPT2 levels are increased in patients with chronic kidney disease [13], increasing in a step-wise fashion with decreasing GFR. To exclude this bias, we included only patients with eGFR > 60 mL/min/1.73 m^2^, so it is highly improbable that the increases in AGPT2 and syndecan-1 are secondary to severe impairment. In this group of patients with reduced GFR, AGPT2 is associated with albuminuria and microinflammation [14]. AGPT2 levels have been evaluated in several groups of patients with renal disease: Anti-Neutrophilic Cytoplasmic Autoantibody (ANCA)-associated vasculitis [15], diabetic nephropathy [16], and lupus [17]. Although AGPT2 has been implicated in endothelial glomerular protein permeability [18], no study has evaluated AGPT2 levels in patients with primary glomerulopathy and near-normal GFR. Other angiopoietin-like proteins are associated with renal fibrosis [19], proteinuria in minimal change disease [20] and proteinuria in kidney allograft recipients [21]. 

It is largely known that nephrotic patients have high cholesterol levels and are susceptible to developing atheromatous disease [22]. Endothelial dysfunction is highly prevalent in nephrotic patients and it is probably related to atherosclerotic changes [23]. Previously, we demonstrated that endothelial glycocalyx injury has an important association with endothelial dysfunction when measured by flow-mediated dilatation in nephrotic patients [3]. In this study we demonstrate that although syndecan-1 is independently associated with LDL-cholesterol when we control for several clinical and laboratory parameters, when mediation analysis was performed, there was no significant direct effect of LDL-cholesterol on syndecan-1, and AGPT2 explained 56% of the total effect of LDL-cholesterol on syndecan-1 levels.

Due to the limitations of mediation analysis (described in limitations), our study can only suggest that AGPT2 mediates endothelial glycocalyx breakdown in nephrotic patients; however, recent evidence using confocal and atomic force microscopy concluded that exogenous AGPT2 induces a rapid loss of the glycocalyx in endothelial cells in vitro [24]. Considering all data together, we speculate that hypercholesterolemia, mainly LDL-cholesterol, leads to glycocalyx derangement through increments of AGPT2. Subsequently, as demonstrated by our group [3], glycocalyx injury leads to endothelial dysfunction, and together, contribute to atheromatous disease.

There are several limitations to our study. First, the relative low number of included patients. Second, although we tried to select individuals in the control group that matched the patient’s main characteristics, it was difficult to select young individuals with no comorbidities and even minor reductions in the eGFR. So, although unlikely, we cannot rule out that difference in syndecan-1 and AGPT2 levels were not due to eGFR differences between patients and control. Third and perhaps the most importantly, due to its cross-sectional design, this study cannot demonstrate causality. Mediation analysis provides evidence supportive of potential causal pathways that must then be confirmed by appropriate interventional studies in animal models or clinical trials. Also, although we adjusted the models for several known potential confounders, it is possible that the described effects may be attributable to unknown variables. For example, we did not evaluate angiopoietin-like 4 that links proteinuria with hypertriglyceridemia in nephrotic syndrome [25]. Finally, because we did not have follow-up data on these patients, is not possible to evaluate if AGPT-2 and syndecan-1 returned to basal levels after nephrotic syndrome remission. 

## 5. Conclusions

Our study suggests that urinary protein excretion is associated with higher LDL-cholesterol and, subsequently, glycocalyx derangement is possibly mediated by AGPT2.

## Figures and Tables

**Table 1:** Clinical and biomarker characteristics of control subjects and patients.

	Glomerulopathy Patients (*n* = 65)	Controls (*n* = 25)	*p*
Age (years)	38.1 ± 12.6	38.5 ± 9.6	0.830
Gender (M/F)	28/37	11/14	1.000
Renal biopsy diagnosis			
*FSGS/ML*	32		
*MN*	18		
*IgAN*	09		
*MPGN*	04		
Serum creatinine (mg/dL)	1.1 ± 0.2	0.82 ± 0.1	<0.001
Serum urea (mg/dL)	43.4 ± 28.7	36.2 ± 11.2	0.227
GFR (ml/min/1.73 m^2^)	87.3 ± 19.6	107.4 ± 9.6	<0.001
Serum albumin (g/dL)	2.6 ± 0.9	4.06 ± 0.32	<0.001
Total cholesterol (mg/dL)	297.6 ± 106.3	183.4 ± 26.7	<0.001
LDL cholesterol (mg/dL)	181.4 ± 71.1	86.0 ± 16.5	<0.001
HDL cholesterol (mg/dL)	56.2 ± 28.1	50.6 ± 9.5	<0.001
Triglycerides (mg/dL)	261.3 ± 108.1	134.7 ± 27.0	<0.001
24 h urinary protein excretion (g/1.73 m^2^)	5.9 ± 2.2	0.08 ± 0.01	<0.001
Angiopoietin-2 (pg/mL)	7505 ± 1354	862 ± 101	<0.001
Syndecan-1 (ng/mL)	102.8 ± 36.2	28.2 ± 9.8	<0.001

FSGS: Focal and segmental glomerulosclerosis; ML: Minimal lesions; MN: Membranous Nephropathy; IgAN: IgA nephropathy; MPGN: Membranoproliferative Glomerulonephritis; GFR: Glomerular filtration rate; LDL: Low density lipoprotein; HDL: High-density lipoprotein.

**Table 2:** Partial correlations (adjusted for age and glomerular filtration rate) between endothelial/glycocalyx biomarkers and nephrotic syndrome features.

	Serum Albumin	Total Cholesterol	LDL Cholesterol	HDL Cholesterol	Triglycerides	24 h Urinary Protein Excretion
Syndecan-1	−0.039	0.282	0.388	0.306	0.261	0.260
	*p* = 0.828	*p* = 0.005	*p* < 0.001	*p* = 0.014	*p* = 0.037	*p* = 0.038
Angiopoietin-2	−0.256	0.298	0.408	0.051	0.111	0.332
	*p* = 0.047	*p* = 0.016	*p* = 0.003	*p* = 0.690	*p* = 0.382	*p* = 0.007

LDL: Low density lipoprotein; HDL: High-density lipoprotein.

**Table 3:** Multivariate analysis to determine the association of clinical and nephrotic syndrome features with syndecan-1.

Variable	Standardized β Coefficient	*p*
Age (years)	1.158	0.041
GFR (mL/min/1.73 m^2^)	−0.126	0.556
Serum albumin (g/dL)	−0.204	0.263
Total cholesterol (mg/dL)	0.139	0.508
LDL-cholesterol (mg/dL)	0.469	0.001
HDL-cholesterol (mg/dL)	0.265	0.167
Triglycerides (mg/dL)	0.192	0.321
24 h urinary protein excretion (mg/1.73 m^2^)	0.193	0.632

GFR: Glomerular filtration rate; LDL: Low-density lipoprotein; HDL: High-density lipoprotein.
